# Arterial compliance is improved via enteral serine protease inhibition in experimental trauma/hemorrhagic shock

**DOI:** 10.3389/fphys.2025.1655946

**Published:** 2025-08-12

**Authors:** Joyce B. Li, Fernando dos Santos, Cynthia R. Muller, Nathalia J. D. Moreira, Luciano F. Borges, Maria Claudia C. Irigoyen, Geert W. Schmid-Schönbein, Erik B. Kistler

**Affiliations:** ^1^ Shu Chien-Gene Ley Department of Bioengineering, University of California, San Diego, La Jolla, CA, United States; ^2^ Department of Anesthesiology and Critical Care, University of California, San Diego, La Jolla, CA, United States; ^3^ Instituto do Coração, Hospital das Clínicas, Faculdade de Medicina, Universidade de São Paulo, São Paulo, Brazil; ^4^ Laboratory of Molecular and Experimental Pathology, Federal University of São Paulo, São Paulo, Brazil; ^5^ Veterans Affairs San Diego Healthcare System, San Diego, CA, United States

**Keywords:** trauma, hemorrhagic shock, arterial compliance, diastolic pressure, protease inhibition, circulatory shock, resuscitation

## Abstract

**Background:**

Systemic hypotension remains a challenge in trauma/hemorrhagic shock (T/HS). Despite intensive vasopressor and fluid therapy, mean arterial blood pressure (MAP) may become refractory to treatment. Arterial compliance (AC) is a critical determinant of arterial hemodynamics but is often overlooked in acute shock states. Considering previous findings on the benefits of enteral protease inhibition in preserving vascular resistance after T/HS, this study investigated both the role of AC and the effects of enteral protease inhibition on AC in T/HS.

**Methods:**

Wistar rats underwent experimental T/HS by laparotomy and exsanguination to induce a MAP of ∼40 mmHg for 90 min. Animals were randomized into three groups corresponding to the intervention: shed whole blood (WB), Lactated Ringer’s (LR), and LR with enteral gabexate mesilate treatment (LR+GM). Resuscitation (120-min period) was initiated by fluid reperfusion with a goal MAP of 65 mmHg. AC was measured via pulse wave velocity (PWV), passive pressure myography, and atomic force microscopy (AFM), with healthy donor arteries for comparison.

**Results:**

PWV increased by ∼15% in all groups after shock. After resuscitation, LR-only animals maintained high PWVs, but significantly lower diastolic pressures (27 mmHg) compared to GM-treated (37 mmHg; p < 0.05) and those reperfused with WB (52 mmHg; p < 0.01). T/HS arteries, particularly the untreated LR arteries, exhibited leftward shifts in circumferential tension-strain curves. LR arteries exhibited higher tangent moduli (5 N/m; p < 0.01) at low physiological stresses, which was corroborated by reduced opening angles, increased mechanical stiffness, alterations in the extracellular matrix, and increased MMP/elastase-like activity. LR+GM and WB arteries displayed elastic moduli and vascular structures more similar to those of healthy arteries.

**Conclusion:**

Experimental T/HS results in impaired AC, which is partially attenuated by enteral GM administration. Vascular biomechanical impairment may underlie the unrestored MAP in fulminant shock. By targeting modulators of AC, with enteral serine protease inhibition as an adjunct intervention, hemodynamic stability and patient outcomes may be improved in T/HS.

## 1 Introduction

Trauma is a serious global health issue as it remains the primary cause of death among individuals under 44 years old (GBD, 2017 Causes of Death Collaborators, 2018; Heron, 2021), with the majority of preventable deaths attributed to post-injury hemorrhagic shock (Eastridge et al., 2019). Hemodynamic instability is a major challenge in trauma/hemorrhagic shock (T/HS) management and is manifested clinically in systemic hypotension and poor organ perfusion. The standard resuscitation strategy for T/HS involves whole blood (WB) or blood product transfusions to replenish lost volume and oxygen carrying capacity (Bouglé et al., 2013; Chipman et al., 2020; Jones et al., 2021). Although WB and blood products have been associated with favorable outcomes (Watts et al., 2022; Shackelford et al., 2017; Braverman et al., 2021), they are not ubiquitously available and affordable, and in this eventuality, crystalloid solutions are routinely administered as fluid replacement alternatives. Vasoconstrictive agents, like norepinephrine or vasopressin, are also administered to raise blood pressure by increasing afterload and systemic vascular resistance (SVR) (Bouglé et al., 2013). However, patients in severe T/HS may become refractory to both fluid therapy and vasopressor intervention and sustain intractably low blood pressures (Valverde, 2021).

Patients with acute hypovolemia or ischemia can also develop a “leaky gut”, or impairment of intestinal wall integrity (Alsaigh et al., 2015; Sertaridou et al., 2015). Disruption of the bowel mucosal barrier allows enteral proteases and gut-/proteolytically-derived moieties to pass into the circulation, where they can potentially trigger adverse vasoactive or inflammatory responses that worsen the shock condition (Chang et al., 2012; Kistler et al., 2012; Li et al., 2019). By neutralizing the pathological mediators directly at the source, our group has previously shown that enterally-delivered serine protease inhibitors in experimental T/HS improve hemodynamics and survival through the preservation of SVR and autonomic function (Santamaria et al., 2017; Aletti et al., 2022; Dos Santos et al., 2022). Enteral protease inhibition can increase mean arterial blood pressure (MAP) after T/HS with crystalloid reperfusion (Dos Santos et al., 2022), but is unable to restore baseline MAP values unless reperfusion includes WB or blood components (Santos et al., 2022; Li et al., 2020). Based on prior observations, we hypothesize that the inability to recover pre-hemorrhage MAP in the absence of WB resuscitation may largely be due to the limited improvement of the diastolic arterial blood pressure (DAP) (Dos Santos et al., 2022; Santos et al., 2022; Li et al., 2020).

From an engineering perspective (as summarized in the Windkessel theory), the maintenance of blood pressure during diastole is predominantly governed by systemic vascular resistance and total arterial compliance (Westerhof et al., 2009). Vascular resistance, which is mainly determined by blood vessel radius, is regularly targeted clinically through the prominent use of vasopressors; but arterial compliance (AC), or the ability of the vascular wall to expand and recoil in response to pulsatile changes in blood pressure, is often neglected in T/HS, despite its significant contribution to DAP–and ultimately, MAP. Any alterations to the geometry or structural components of large arterial vessels may affect AC or elastance, and in turn, impair vascular hemodynamics (Butlin et al., 2020; Cocciolone et al., 2018). Because the functional status of large artery compliance in hypovolemic shock is yet to be resolved, we sought to investigate the effects of fluid-resuscitated T/HS on AC and to determine the impact of enteral serine protease inhibition on AC in our experimental T/HS model.

## 2 Materials and methods

### 2.1 T/HS experimental design and procedure

Twenty-eight male Wistar rats (360–460 g, 3–4 months, Charles River Laboratories) were randomized into four groups (n = 7/group), corresponding to the resuscitation interventions received after T/HS [Lactated Ringer’s only (LR), Lactated Ringer’s with enteral serine protease inhibitor gabexate mesilate (LR+GM), or shed whole blood (WB)] or no-T/HS no-intervention (Healthy). Animals were anesthetized with 5% isoflurane in an isolated chamber for induction and with 1.5% isoflurane delivered by nose cone for maintenance at a flow rate of 0.8 L/min. The right femoral artery and the left carotid artery were cannulated with equal-length catheters (PE 10 tubing) for continuous blood pressure and heart rate monitoring at 2 kHz/channel by PowerLab^®^ paired with LabChart 7.0 (ADInstruments, Dunedin, NZ). The right femoral vein was cannulated (PE 50 tubing) for heparinization (100 units/kg), blood withdrawal, and intravenous (IV) fluid therapy. Animals were placed on a water-heated platform to maintain body temperature at 37°C, monitored with a rectal probe for the duration of the experiment.

Abdominal trauma was carried out via midline laparotomy and a double-lumen enteral catheter was orally inserted with the tip placed within the proximal duodenum, as previously described (Dos Santos et al., 2022). All animals subjected to T/HS were enterally infused with the vehicle solution Golytely^®^ (0.14 g/mL sterile water, 100 μL/min for 150 min) at 20 min into the shock period. Gabexate mesilate (10 mg/kg) was added to the vehicle solution of the treated group, LR+GM.

After the midline incision was closed with running suture, a 20-min period of baseline stabilization was initiated. Hemorrhagic shock was then induced by controlled exsanguination (0.5 mL/min) to maintain a MAP of 35–40 mmHg for 90 min. The hypotensive period was followed by IV fluid resuscitation at 2 mL/min with either warmed Lactated Ringer’s solution with 5% dextrose or warmed shed whole blood to target a hemodynamic goal of MAP ≥ 65 mmHg. Animals were monitored and reperfused with the necessary volume to maintain a stable MAP for 120 min, before subsequent termination (B-Euthanasia, 120 mg/kg) and adjunctive bilateral thoracotomy.

The left femoral artery and thoracic aorta were excised from T/HS animals and healthy donor animals immediately upon euthanasia for biomechanical testing, histology, and other assays. Half of the aortic tissue was flash-frozen in liquid nitrogen and stored at −80°C, and the other half was submerged in 10% formalin for 24-h fixation.

Animal care and handling were in compliance with the NIH Guide for Care and Use of Laboratory Animals and the animal protocol (S16062) was approved by the Institutional Animal Care and Use Committee of University of California, San Diego.

### 2.2 Laboratory blood tests

Blood samples were drawn at baseline, the end of the hypotension period (Shock), 30 min (r30) and 120 min (r120) after the start of reperfusion for laboratory tests. Hemoglobin was measured using the Hb 201+ spectrophotometer system (HemoCue^®^, Brea, CA). Arterial blood gas analysis (ABL90 FLEX, Radiometer Medical, Brea, CA) was immediately performed upon collection for measurement of the following parameters: pH, oxygen partial pressure (PO_2_), carbon dioxide partial pressure (PCO_2_), bicarbonate (HCO_3_), base excess (BE), oxygen saturation (O_2_ sat) and glucose levels. Blood lactate concentration was measured using an electrochemical analyzer (Lactate Plus Meter, Nova Biomedical, Waltham, MA).

### 2.3 Pulse wave velocity measurement

To directly measure changes in AC *in vivo*, the transit time for the pressure wave to propagate down the aorta was measured by the time delay between the foot of the two simultaneous waveforms from the carotid- and femoral-accessed arterial signals. The distance between the two catheter tips were post-operatively marked and measured. The carotid-femoral pulse wave velocity (PWV) was then determined by dividing the measured distance by the pulse wave transit time, averaging the PWV for 18 consecutive cardiac cycles at each major timepoint of the experiment for sufficient representation of the timepoint.

### 2.4 Exponential curve fitting for tau estimation

According to the two-element Windkessel model, which parallels the arterial system to a simplified RC circuit, diastolic decay time constant (τ) corresponds to the product of SVR and AC, assuming steady-state pressure and flow (Westerhof et al., 2009). The time constant τ was estimated by fitting a one-phase decay exponential curve [ $P\left(t\right)=\left(P_{0}-P_{asymp}\right)*e^{-t/\tau }+P_{asymp}$ , where P_0_ is the pressure at end-systole and P_asymp_ is the theoretical pressure that plateaus at infinite time] to the diastolic period of the femorally-accessed central waveform using GraphPad Prism 7 (GraphPad Software, San Diego, CA). The reported τ values were obtained by averaging 6 consecutive time constants evaluated for goodness-of-fit at each selected timepoint for sufficient representation of the timepoint.

### 2.5 *Ex vivo* passive pressure myography

Excised femoral arteries from T/HS animals and healthy animals were submerged in a Ca^2+^-free Krebs-Henseleit solution (37°C, pH = 7.4, 95% O_2_/5% CO_2_ aeration) and cannulated with glass micropipettes inside a servo-controlled pressure-regulated myography chamber (Living Systems Instrumentation, St Albans, VT) for biomechanical testing under passive conditions. Each blood vessel was stretched to its pre-recorded *in vivo* length and was allowed to stabilize for 30 min before undergoing four cycles of preconditioning, where intraluminal pressure was increased and decreased from 0 mmHg to 180 mmHg in 15 mmHg increments for 30-s holds. Preliminary tests indicated a consistent and repeatable response after the fourth cycle, validating the use of the fifth loading and unloading cycle for mechanical characterization. The arteries were visualized under a camera-equipped microscope (×10 objective), and real-time vessel diameters were measured using VasoTracker 1.0.3 (Lawton et al., 2019). The axial stretch ratio (λ) was recorded as the ratio between the marked *in vivo* axial length and the no-stress *ex vivo* axial length. Due to the wall thickness of the femoral artery, the inner diameter was unable to be accurately detected; therefore, circumferential tension was calculated using the outer diameter and the incompressibility assumption: $T_{\theta }=P_{\mathrm{int}}*\sqrt{\left(r_{ext}^{2}-\frac{R_{ext}^{2}-R_{\mathrm{int}}^{2}}{\lambda }\right)}$ , where P_int_ = internal pressure, r_ext_ = loaded external radius, R_ext_ = no-load external radius, R_int_ = no-load internal radius. Circumferential strain was calculated as such: $\varepsilon_{\theta }=\frac{\left(D_{p}-D_{0mmHg}\right)}{D_{0mmHg}}$ , where D_p_ = outer diameter at a specific pressure and D_0mmHg_ = outer diameter at zero internal pressure. Energy loss percentage within the tissue was calculated from normalizing the total hysteresis loop area (energy dissipated) by the integral of the loading stress-tension curve (total energy absorbed). Tangent moduli of the lower and higher linear regions of the stress-tension curve were derived from the slopes of the linear regressions of the first and last four data points, respectively, as another method to describe material stiffness. The incremental elastic moduli of the physiological pressure range were also determined by estimating the tangent slopes along the polynomial fit of the curves. One artery per group was excluded in analysis because the no-load diameters were not recorded.

### 2.6 Atomic force microscopy (AFM) imaging of aortic tissue

Formalin-fixed aortic tissues were embedded in paraffin and cut into 5-μm sections before being mounted and dried on glass slides. The tissue sections were deparaffinized and rehydrated in phosphate-buffered saline (PBS) prior to mechanical characterization (n = 4/group). The tissues were scanned in PBS using a Nanowizard^®^ 4a BioScience AFM (JPK/Bruker, Germany) with a pre-calibrated cantilever (spring constant ∼0.3 N/m, 1 µm tip radius; Bruker, Germany) on force mapping mode (Z length = 1.5 µm, setpoint = 12 nN, scan rate = 1 μm/s). Random 40 × 40 μm^2^ areas (8 × 8 pixels) of the tunica media were imaged in triplicates for every sample. Force-distance curves were fitted and analyzed to estimate the average Young’s modulus. Relative elastic moduli (versus true intrinsic values) were determined as sample preparation may augment tissue stiffness.

### 2.7 Opening angle measurement of aortic ring

Flash-frozen aortic vessels were thawed in 37°C Krebs-Henseleit solution, sliced into 1 mm-thick rings, and allowed to stabilize in the solution for 15 min. Afterwards, the rings were radially cut and allowed to stabilize for another 15 min. The open sectors were then imaged and the opening angle (the angle formed by cut ends and the midpoint of the circular arch) were measured as an indicator of circumferential residual stress within the aorta wall.

### 2.8 Histological analysis and multiphoton imaging of aortic tissue

5-μm sections of fixed aortic tissues were deparaffinized and rehydrated prior to histological staining. Elastic components (fibers and elastic lamellae) were identified using Verhoeff’s and Miller’s staining method, while collagen fibers were stained using the Picrosirius method with counterstaining of nuclei by hematoxylin (De Figueiredo et al., 2008). The stained sections were dehydrated, cleared, and mounted for visualization. An upright Nikon Eclipse 80i microscope, equipped with a polarizer and a color camera (FLIR, Wilsonville, OR), was used to image the slides. Histological evaluations were performed by a secondary investigator and a clinical pathologist blinded to the study groups, using ImageScope software (Leica Biosystems, Vista, CA). Multiphoton microscopy of elastin autofluorescence and collagen second harmonic generation signals at ×40 magnification (water immersion) were used to confirm histological findings.

### 2.9 Gelatin zymography on plasma and aortic tissue homogenates

Plasma from all four sampling timepoints were diluted 1:5 with PBS. Frozen aortic tissues were ground with a pellet pestle in liquid nitrogen and homogenized in 100 μL RIPA lysis buffer with EDTA-free protease inhibitor cocktail (HALT™, ThermoFisher) before centrifugation at 16,000 *g* and 4°C for 20 min. Supernatant was collected and aliquoted for protein quantification using a colorimetric assay (Pierce™ BCA Kit, ThermoFisher). Equal volumes of diluted plasma (4 µL) and equal amounts of protein (40 μg) from tissue homogenates were loaded into individual wells of 10% Tris-Glycine gels with 0.1% gelatin (Invitrogen, Carlsbad, CA). Pre-stained molecular weight standard (Precision Plus Protein™, Bio-Rad, Irvine, CA) and recombinant MMP-9 (Sigma-Aldrich) were loaded into each gel as references. After electrophoresis, the proteins were renatured with Triton X-100 buffer for 30 min and then activated in developing buffer (Invitrogen) overnight in 37°C. As a negative control to verify for MMP-specific activity, gels were incubated in developing buffer supplemented with 20 mM EDTA. Gels were stained with 0.1% Coomassie Blue R-250 in 40% methanol/10% acetic acid and destained until clear bands were detected for imaging and analysis via ImageJ/Fiji software.

### 2.10 Serine protease enzymatic activity of aortic tissue homogenates

Enzymatic activity was measured using fluorogenic peptide substrates for trypsin-like activity (Z-Arg-Arg-Leu-Arg-AMC) and elastase-like activity (MeOSuc-Ala-Ala-Pro-Val-AMC). A 384-well plate was utilized to mix 15 μL of aorta homogenate (without protease inhibitor cocktail, diluted 1:10 in PBS, pH = 7.4) with 15 μL of substrate (10 μM in PBS) in triplicates. The protease activity of the mixture was immediately assessed using a spectrophotometer (FilterMax F5 Multi-Mode, Molecular Devices) with excitation wavelength at 360 nm and emission at 460 nm. Readings were taken every 135 s over a duration of 3 h and 45 min, resulting in fluorescent activity progress curves comprising of exactly 100 data points each. Fluorogenic activity was quantified as relative fluorescence units per second, using a region of the progress curve with a slope higher than *r* ^2^ = 0.98. The activity was adjusted for the dilution factor and total protein in the sample.

### 2.11 Statistical analysis

Data were analyzed using GraphPad Prism 10 and are presented as mean ± SEM unless indicated otherwise. Linear regression analyses were performed to evaluate the correlation between blood pressure and aortic PWV. One-way or two-way analysis of variance (ANOVA) was applied where appropriate to evaluate differences between groups and timepoints. *Post hoc* analyses were performed with Tukey’s multiple comparisons test. Results were interpreted as significant if p-value < 0.05.

## 3 Results

### 3.1 Hemodynamics

**FIGURE 1:**
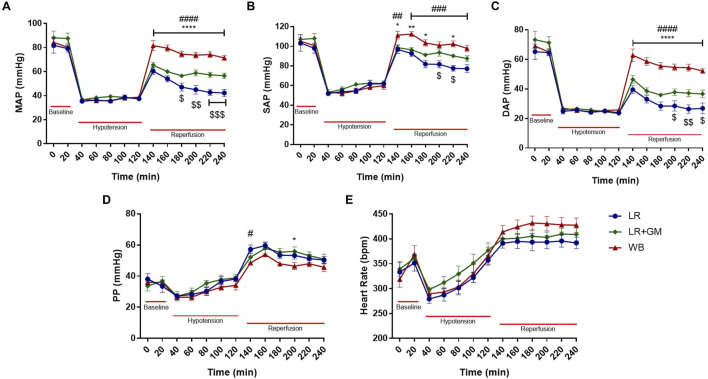
Hemodynamics. Temporal analysis of mean **(A)**, systolic **(B)**, diastolic **(C)**, and pulse **(D)** arterial blood pressure and heart rate **(E)** shown as mean ± SEM span from baseline to the end of 2-h reperfusion following 90-min hypotensive shock. $ p < 0.05, $$ p < 0.01, and $$$ p < 0.001 for LR vs. LR+GM, # p < 0.05, ## p < 0.01, ### p < 0.001, and #### p < 0.0001 for LR vs. WB, and * p < 0.05, ** p < 0.01, and **** p < 0.0001 for LR+GM vs. WB at the same timepoint, using two-way ANOVA.

The vital signs tracings of the LR and LR+GM groups paralleled the results from our previous study (Dos Santos et al., 2022), validating the hemodynamic advantages of enteral GM treatment (Figure 1). With no differences in body weight between groups, the GM-treated group required less resuscitative volume to maintain MAP stability, had higher hemoglobin levels, and had lower lactate levels than the LR-only group (complete table of laboratory parameters is available in the Supplementary Material). Despite the notable improvements in blood pressure compared to the untreated LR group, hemodynamic recovery in the LR+GM group was inferior compared to animals reperfused in WB (positive control for optimal resuscitation), particularly the DAP (Figure 1C). Pulse pressure (PP) in all groups gradually increased during the shock period and immediately upon fluid resuscitation (reperfusion), after which both LR-resuscitated groups had relatively higher PP values than the WB group until termination of the experiment (Figure 1D). No statistically significant differences in heart rate were observed between the groups.

### 3.2 *In vivo* estimations of arterial compliance and diastolic time constant (τ)

**FIGURE 2:**
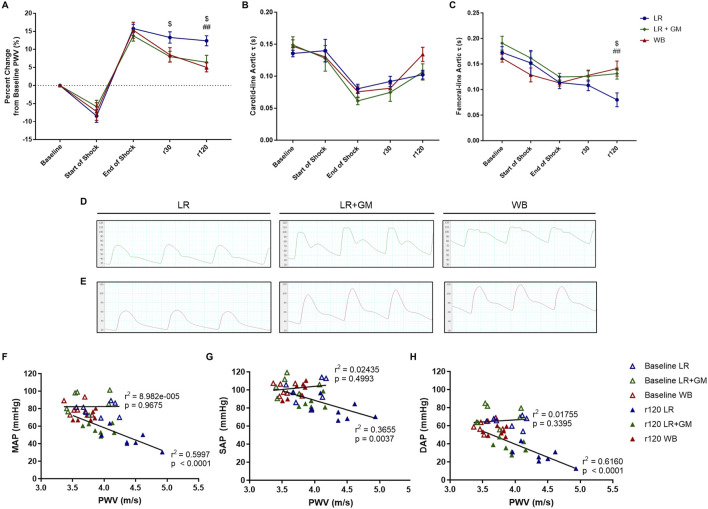
*In vivo* aortic pulse wave velocity (PWV) and diastolic τ measurements. The percent change from baseline levels of carotid-femoral PWV **(A)**, the aortic diastolic τ accessed from the carotid tracing **(B)**, and the aortic diastolic τ accessed from the femoral tracing **(C)** were calculated from continuous arterial blood pressure waveforms of LR (blue), LR+GM (green), and WB (red) animals. Examples of the waveforms from the carotid measurements **(D)** and femoral measurements **(E)** at r120 (end of experiment) from each group are presented. Correlation analysis between PWV and MAP **(F)**, SAP **(G)**, and DAP **(H)** was conducted for values at baseline (open triangles) and at r120 (filled triangles). Data are presented as mean ± SEM. $ p < 0.05 for LR vs. LR+GM, ## p < 0.01 for LR vs. WB at the same timepoint, using two-way ANOVA.

For all T/HS groups, PWV decreased at the start of shock induced by exsanguination, but by the end of shock after 90 min of sustained hypotension, PWV was increased by ∼15% from baseline PWV (Figure 2A). At timepoints r30 and r120, the untreated LR animals continued to present elevated PWV, while both LR+GM and WB animals were able to significantly lower PWV closer to baseline values.

Conversely, the diastolic time constant τ was decreased at the end of the shock period by over 30% when compared to baseline values (Figures 2B,C). A divergence between the groups was observed after 30 min into resuscitation in the femorally-accessed aortic waveforms, as the average estimated τ of the LR group drastically declined while that of the LR+GM and of the WB group slightly increased. Representative of the distinct diastolic decay behaviors quantified in Figures 2B,C, visualizations of the blood pressure waveforms at the end of resuscitation (r120) from each group (LR, LR+GM, WB) are depicted in Figures 2D,E. Linear correlation analysis (Figures 2F–H) revealed a strong negative correlation between PWV and blood pressure after T/HS and fluid reperfusion, particularly with DAP vs. PWV (*r* ^2^ = 0.616).

### 3.3 *Ex vivo* passive pressure myography measurements of T/HS femoral arteries

**FIGURE 3:**
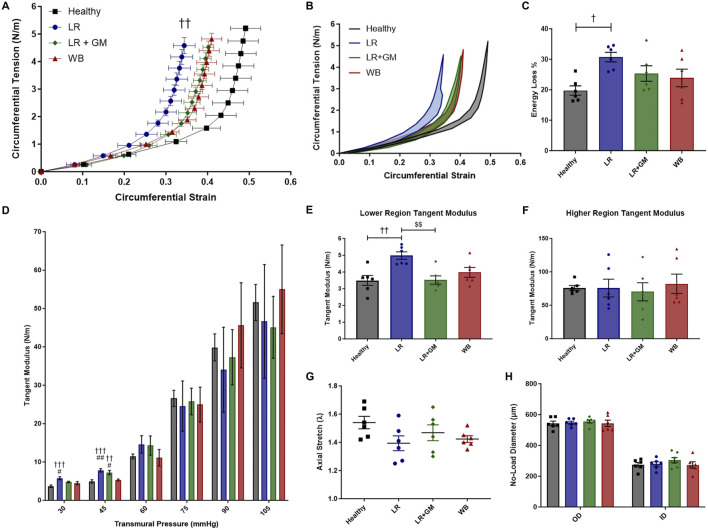
*Ex vivo* passive pressure myography. Biomechanical testing of excised femoral arteries from healthy control (black), untreated LR-resuscitated T/HS (blue), GM-treated LR-resuscitated T/HS (green), and WB-resuscitated T/HS (red) animals generated the following data: circumferential tension-strain loading curves **(A)**, average hysteresis areas **(B)**, energy loss percentages **(C)**, tangent moduli at physiological pressures **(D)**, tangent moduli of the lower linear region **(E)** and higher linear region **(F)**, and axial stretch ratios **(G)**. The outer and inner diameters **(H)** at zero-load were also measured, indicating no differences in wall thickness between groups. Data are presented as mean ± SEM. By one-way ANOVA, † p < 0.05, †† p < 0.01, and ††† p < 0.001 for Healthy vs. LR or LR+GM, # p < 0.05 and ## p < 0.01 WB vs. LR or LR+GM, and $$ p < 0.01 for LR vs. LR+GM.

All groups that underwent T/HS displayed a leftward shift in their circumferential tension-strain curves, especially the untreated LR group, compared to the Healthy control group, indicating less deformation or distensibility for a given load (Figure 3A). The average energy loss and tangent moduli at lower pressures (DAP range) and of the lower linear region were also significantly higher in the LR group compared to the Healthy non-shock group (Figures 3B–E). Enteral GM treatment was able to partially prevent these biomechanical changes, as LR+GM arteries, comparable to WB-resuscitated arteries, showed less pronounced shifts and lower initial slopes in tension-strain curves than LR-only arteries. No statistically significant differences were found between the groups on the tangent modulus of the higher linear region, the longitudinal stretch ratio (p < 0.13 Healthy vs. LR), and no-load diameters of the vessels (Figures 3F–H).

### 3.4 AFM analysis of T/HS aortic tissue

**FIGURE 4:**
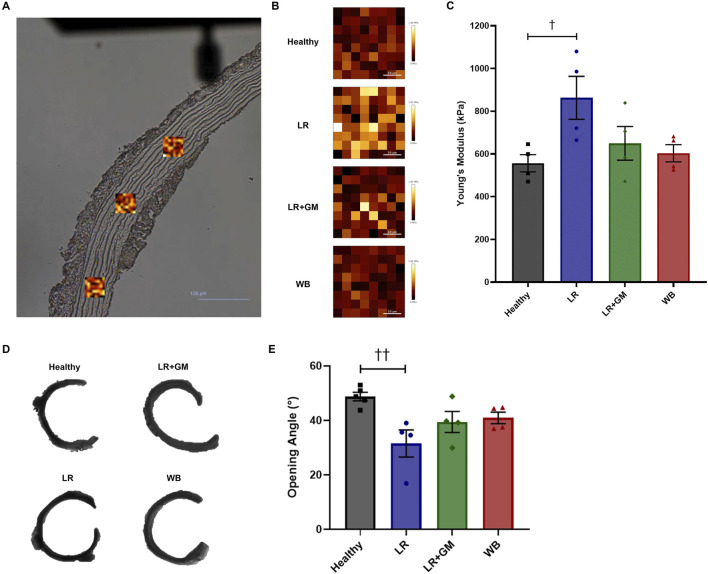
Force mapping via atomic force microscopy and opening angle measurements of healthy and T/HS aortic rings. **(A)** Areas (scan size: 40 μm) of the tunica media were scanned in triplicates per aortic section (scale bar = 100 μm). **(B)** Representative scans of Young’s modulus mapping from each group were set to the same color scale (0–1.92 MPa) for visual comparison. **(C)** The mean of the average Young’s modulus of each sample scan was calculated and plotted on a bar graph. **(D)** Representative images of cut-open aortic rings from each group. **(E)** Average opening angles measured in degrees. Data are presented as mean ± SEM. By one-way ANOVA, †† p < 0.01 and † p < 0.05 for Healthy vs. LR.

After averaging the mean Young’s moduli per sample, we found that the aortic media of the untreated LR group was significantly stiffer than that of the healthy group (862.7 vs. 556.5 kPa, p < 0.0486) (Figure 4C). The LR+GM and WB aortic tissues had Young’s modulus values slightly higher than the healthy tissues but not statistically different. Representative scans set to the same color scale (0–1.92 MPa) are depicted for relative visual comparison (Figure 4B).

### 3.5 Opening angle analysis of T/HS aortic rings

Sectors of untreated LR aortas had markedly smaller opening angles compared to those of Healthy aortas (31.5° vs. 48.7°, p < 0.01) (Figures 4D,E). GM-treatment and WB reperfusion were able to slightly improve the aortic opening angle with averages around 40° for both.

### 3.6 Histological analysis of T/HS aortic tissue

**FIGURE 5:**
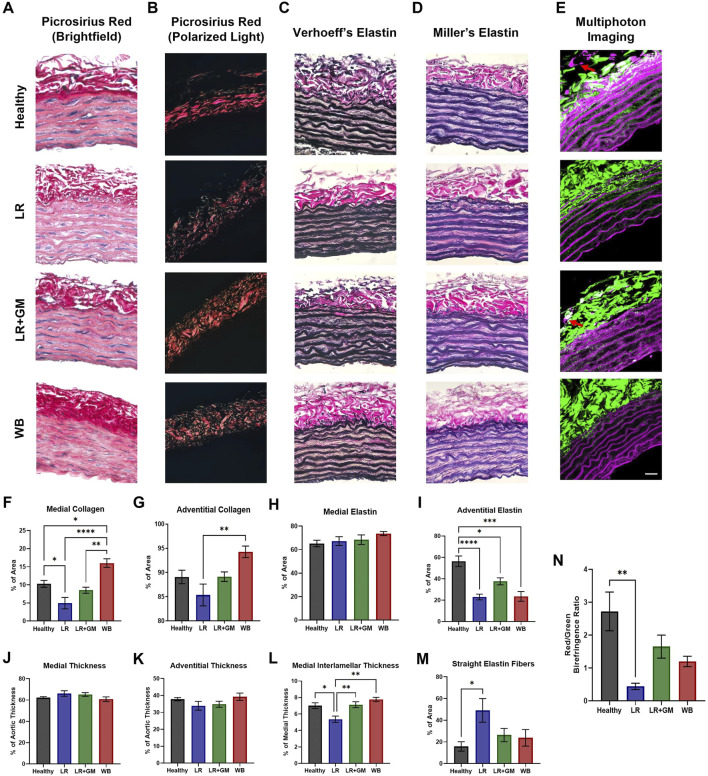
Histological analysis of extracellular matrix changes in healthy and T/HS aortic tissue. Representative images of histological staining using picrosirius/hematoxylin under **(A)** brightfield & **(B)** polarized light, **(C)** Verhoeff’s staining method, **(D)** Miller’s staining method in healthy donor aorta and T/HS aortic tissues resuscitated with LR, LR+GM, and WB are shown. **(E)** Representative images of elastic fibers (magenta) via two-photon excitation fluorescence and collagen fibers (green) via second-harmonic generation of Healthy, LR, LR+GM, and WB vascular tissue were generated. The percentages of collagen **(F, G)** and elastin **(H, I)** in the aortic media and adventitia were quantified, along with the thickness of the medial **(J)**, adventitial **(K)**, and interlamellar **(L)** layers. Percentage of straight (non-undulated) elastic fibers in the medial layer was also measured **(M)**, along with the ratio of red to green birefringence collagen fibers in the polarized area **(N)**. Images were taken at ×40 magnification with lumen at the bottom of frame (scale bar = 25 μm). Data are presented as mean ± SEM. By one-way ANOVA, * p < 0.05, ** p < 0.01, *** p < 0.001, and **** p < 0.0001 for the marked groups.

Picrosirius staining revealed a notable reduction in collagen in the medial layer in the untreated LR aortas compared to healthy aortas (4.93% vs. 10.27% of media area, p < 0.05) (Figures 5A,F). Polarized light microscopy demonstrated altered collagen organization in the adventitia of the LR group, with measurably higher proportions of green birefringence, indicative of thinner collagen fibers, and fragmentation (Figures 5B,N). Conversely, GM treatment preserved both medial and adventitial collagen compositions similar to those of healthy controls. Interestingly, WB-reperfused animals had aortas with increased collagen content in both the media (16.0%) and adventitia (94.6% vs. 85.4% of LR group, p < 0.01).

No distinguishable differences were observed in the quantification of elastic components in the medial layer (Figure 5H). However, all T/HS groups displayed a significant decrease in adventitial elastic fibers, with GM treatment providing partial preservation (Figure 5I). Multiphoton microscopy corroborated these findings (red arrows, Figure 5E). Despite similar average medial and adventitial thicknesses across all groups, the LR group exhibited a significant decrease in interlamellar thickness (space between elastin lamellae) compared to other groups (Figures 5J–L). These data coincide with the drastic increase in straight (non-undulated) elastin lamellae observed in the medial layer of LR aortas (Figure 5M).

### 3.7 T/HS plasma and aortic tissue matrix metalloproteinase activity

**FIGURE 6:**
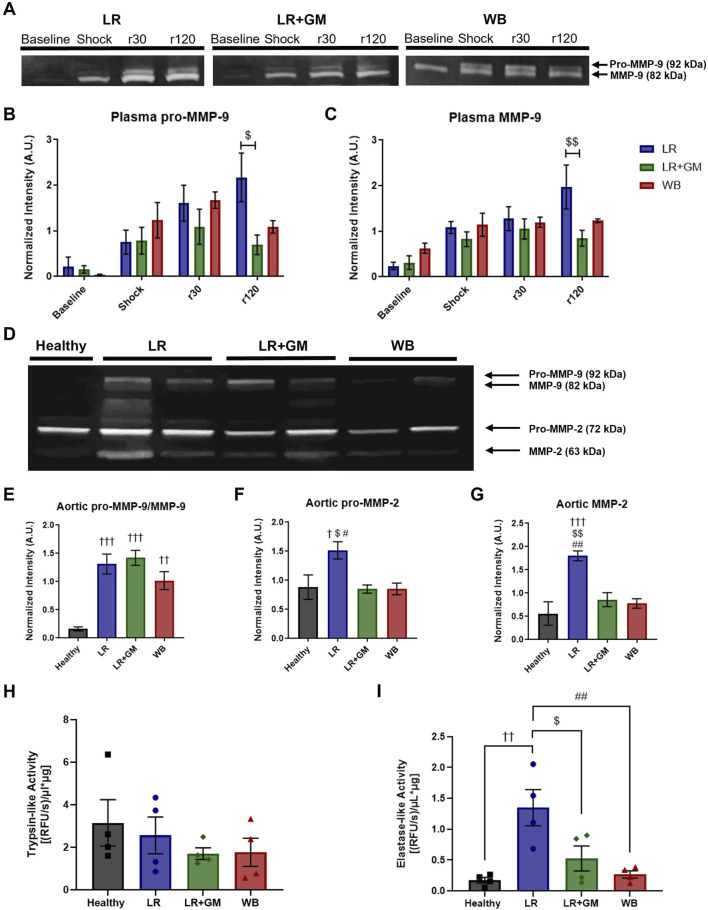
Proteolytic enzyme activity in T/HS plasma and aortic tissue. **(A)** Gelatin zymography of circulating plasma collected from LR (blue), LR+GM (green), and WB animals (red) at specific timepoints (Baseline, Shock (end of shock period), r30, and r120) revealed temporal changes in **(B)** pro-MMP-9 and **(C)** MMP-9. **(D)** Gelatin zymography of aortic tissue homogenates collected at the end of the T/HS experiment was analyzed for relative enzyme activity of **(E)** pro-MMP-9 and MMP-9, **(F)** pro-MMP-2, and **(G)** MMP-2. **(H)** Trypsin-like and **(I)** elastase-like enzymatic activity in aortic tissue homogenates were measured by fluorogenic peptide substrate assays. Data are presented as mean ± SEM. By one-way and two-way ANOVA, $ p < 0.05 and $$ p < 0.01 for LR vs. LR+GM, † p < 0.05, †† p < 0.01, and ††† p < 0.01 for Healthy vs. LR/LR+GM/WB, and # p < 0.05 and ## p < 0.01 for LR vs. WB.

Gelatin zymography experiments detected elevated concentrations of MMP-9 (pro-form and active form) in circulating plasma after T/HS (Figures 6A–C). Plasma from LR-only animals contained increasingly high amounts of MMP-9 activity at r30 and r120, but the addition of enteral GM was able to significantly counteract the escalation. This was not the case within the aortic tissue (Figure 6E), as tissue pro-MMP-9/MMP-9 remained high for all shock groups. GM, along with WB, was however able to prevent elevated activity of MMP-2 in aortic tissue (Figures 6F,G).

### 3.8 Serine protease activity in T/HS aortic wall tissue

The fluorogenic peptide substrate assays showed no differences in trypsin-like activity within aortic tissue between groups (Figure 6H). However, the untreated LR shock group had significantly higher enzymatic velocity with regards to elastase-like activity in homogenized aorta compared to the other groups, including LR+GM and WB (Figure 6I).

## 4 Discussion

In this study, we reaffirmed our previous observations regarding the hemodynamic improvement with enteral GM treatment (Dos Santos et al., 2022), and in addition, employed the WB group as a positive control group (Figure 1). The blood pressure tracings highlight that while systolic blood pressure reached clinically acceptable levels upon fluid resuscitation, DAP remained notably low after LR reperfusion. Consistent with prior T/HS research (Dos Santos et al., 2022; Santos et al., 2022; Moreira et al., 2023; Muller et al., 2021), the depressed DAP appears to be the limiting factor preventing the achievement of adequate MAP. This finding served as the impetus for our investigation into AC, an overlooked element of the Windkessel model in the context of severe hypotension.

A potential indicator of changes to AC is the amplification of central PP during and after shock (Figure 1D), as increased PP (mainly via a decrease in DAP) has been associated with a reduction in AC and changes in ventricular dynamics (Vennin et al., 2017; Elzinga and Westerhof, 1973). Likewise, the rise in PWV following T/HS seen in Figure 2A also suggests decreased AC and increased aortic characteristic impedance (Butlin et al., 2020; Lacolley et al., 2017). Because the MAP was held at ∼40 mmHg throughout the shock period, the ∼20% increase in PWV cannot be directly attributed to a fluctuation in blood pressure, but was rather a consequence of pathophysiological mechanisms caused by prolonged hypovolemia (Nagasawa et al., 2022). This result raises the possibility of a feedback loop, similar to what has been theorized in hypertension (Humphrey et al., 2016), where the sustained pressure drop triggers structural or functional changes to the vasculature, resulting in changes to global hemodynamics. A strong inverse relationship was seen between final PWV and blood pressure values at r120 (Figures 2F–H), especially between PWV and DAP (*r* ^2^ = 0.616). Lower DAP has also been noted in septic shock patients with elevated PWV (Kazune et al., 2019), reinforcing the Windkessel link between AC and DAP.

The decreased diastolic time constant (τ) after shock is also indicative of a reduction in AC and SVR, based on the two-element Windkessel model (RC decay) (Westerhof et al., 2009; Behnam et al., 2019; Vanden et al., 2018). The significant reduction in aortic τ from the femoral line in the LR group is apparent in the blood pressure waveform from the lack of a dicrotic notch and diastolic peak (Figure 2E). The shape of the diastolic run-off can reflect abnormal vascular conditions that disrupt elastic recoil and arterial reservoir pressure (Wang et al., 2003; Manning et al., 2002). While SVR was not calculated in this study, we can infer that it likely decreased after severe T/HS, as our previous studies consistently displayed vasopressor hyporesponsiveness, diminished vascular function in resistance arteries, reduced adrenergic receptor density, and compromised sympathetic function - all of which were able to be partially ameliorated by enteral GM (Dos Santos et al., 2022; Moreira et al., 2023; Mazor et al., 2021). Similar trends of decreased arterial τ, widened aortic PP, decreased SVR, and reduced AC in an experimental septic shock model were reported by Carrara et al., who postulated vascular changes in conduit arteries induced by systemic hypotension and subsequent fluid resuscitation (Carrara et al., 2020). Carrara et al. attributed large artery stiffness and vascular uncoupling in septic shock to nitric oxide bioavailability; however, previous testing of L-NAME in this T/HS model (unpublished data) found no significant hemodynamic differences between treated and untreated groups (Carrara et al., 2020). Altered arterial compliance has also been observed in clinical cases of circulatory shock (Kazune et al., 2019; Nagayama et al., 2019).

However, the active (vascular endothelium/smooth muscle) and passive (extracellular matrix (ECM)) contributions to AC are indistinguishable in *in vivo* measurements. Hence, *ex vivo* biomechanical tests of large arteries were conducted in Ca^2+^-free solution in attempt to isolate the passive structural contributions. Vascular tissues from LR-reperfused T/HS animals exhibited reduced distensibility or deformability, as evidenced by the leftward shifts in the circumferential tension-strain curves and the slightly lower axial stretch ratios when compared to those of healthy tissue (Figure 3). The higher percentage of energy loss in the T/HS arteries also suggests decreased efficiency in elastic recoil during diastole, as more energy was dissipated into the vessel wall (Cocciolone et al., 2018; Gregory et al., 2022). The increased tangent modulus in the lower linear region of the tension-strain curves, often associated with elastin-dominated behavior (Gregory et al., 2022), of the LR group was corroborated by the change in elastin content and lamellae composition revealed by histology and two-photon excitation microscopy (Figure 5). The significant reduction in aortic opening angle after untreated T/HS further supports elastin degradation (Figures 4D,E), as prior research has shown that only elastase or elastin removal was able to significantly decrease the opening angle, while collagenase and vascular SMC removal had minimal effect (Fonck et al., 2007; Greenwald et al., 1997; Giudici et al., 2021). The impaired biomechanics of central arteries due to elastin degradation may help explain the inability to restore DAP and the development of irreversible shock despite volume resuscitation, as elastin fibers cannot regenerate in adult tissue (Yanagisawa and Wagenseil, 2020).

Collagen fibers also undergo changes after T/HS. Collagen degradation was observed in the untreated LR group, evidenced by a decrease in stained collagen area in both the medial and adventitial layers (Figures 5A,F,G) and by the increased presence of green birefringence collagen (Figures 5B,N), indicative of thinner or less crosslinked fibers (Rich and Whittaker, 2017). Additionally, collagen fibers appeared more fragmented after shock compared to healthy controls. Enteral protease inhibition with GM partially mitigated these changes. The increased collagen staining in the WB group, exceeding that of healthy controls, may be due to the uncovering and reorientation of collagen caused by elastin and crosslink degradation, rather than synthesis of new collagen (Figure 5I).

To further confirm the findings of the *ex vivo* viscoelastic changes and the *in vivo* loss in AC, AFM was used to directly measure the Young’s modulus of the aortic samples with high sensitivity. Analysis of the AFM scans revealed that the medial layer of the LR-only T/HS group was indeed more resistant to deformation at nN-range loads than the other groups (Figure 4C). This again may be due to the degradation of interlamellar elastin fibers, which exposes the underlying collagen that is roughly 400× stiffer than elastin (Giudici et al., 2021). The correlation between stiffer arterial tunica media (measured by AFM) and higher PWV has been previously demonstrated in patient samples (Chang et al., 2018) and is in alignment with the observations of this study. Again, enteral GM was able to mitigate the decrease in mechanical compliance, providing a potential explanation for its ability to also preserve baroreflex sensitivity in T/HS, possibly by retaining mechanotransduction capabilities (Dos Santos et al., 2022; Carrara et al., 2020).

The deterioration of large artery ECM components after T/HS may be partially mediated by the increase in proteolytic activity both in the circulation and within the vascular wall (Figure 6). Shock-induced circulating serine proteases, particularly from the gut, may enter the permeable vascular vessel wall from the luminal side after glycocalyx degradation in T/HS (Moreira et al., 2023), but might also penetrate via the vasa vasorum, as suggested by the loss of adventitial elastin in untreated T/HS aortas. Unregulated proteolytic activity within the adventitia suggests possible vulnerability of the autonomic nerves, potentially explaining impaired sympathetic response in shock (Dos Santos et al., 2022). Dysregulated MMP activity has been identified as a deleterious mechanism in various cardiovascular diseases (e.g., aortic aneurysm), involving elastolytic vascular remodeling through vascular inflammation and oxidative stress (Wang and Khalil, 2018). GM may not directly inhibit MMP-2/-9, based on fluorogenic substrate experiments (data not shown), but the serine inhibitor may be decreasing the elevated trypsin-like and elastase-like activity in the systemic circulation after T/HS (Moreira et al., 2023) or the elastase-like activity within the aortic walls after T/HS (Figure 6I), thus decreasing serine protease-induced MMP activation and serine protease-mediated elastin degradation (Cocciolone et al., 2018; Rosário et al., 2004; Heinz, 2020).

Enteral GM has previously been demonstrated to improve SVR (Dos Santos et al., 2022; Moreira et al., 2023), and now AC, but does not completely rescue vascular function (as evidenced by MAP). While the LR+GM group presented compliance behavior comparable to that of WB group, the discrepancy between the two groups in blood pressure suggests that elements within whole blood, but absent in crystalloid solutions, significantly influence hemodynamics. This may be due to differences in fluid shear stress, oxygen carrying capacity, or tissue energetics, as hinted by changes in hemoglobin and lactate levels after shock (Supplementary Table S1). While the chosen dose of GM aimed for optimal enteral serine protease inhibition, it is possible that the protease inhibitor may have a dose-dependent effect, with the current concentration potentially reaching its maximum observable impact on AC. Although serine protease inhibition substantially preserved the integrity of the vascular ECM, the reduced distensibility of LR+GM and WB arteries may have also resulted from the cumulative effects of reperfusion injury (Supplementary Figures S1, S2), inflammatory mediators, reactive oxygen species, or mechanical stress (e.g., >10% cyclic stretch after shock, Supplementary Figure S3) (Lacolley et al., 2017). Comparative analysis with a non-resuscitated T/HS group (ShockNR) suggests reperfusion injury may further exacerbate biomechanical changes observed in all volume-resuscitated shock groups (Supplementary Material), potentially by distributing inflammatory mediators and proteases throughout the circulation and accelerating vascular tissue destruction (Figures 6A–C). These factors may account for the circulatory failure still apparent in all T/HS groups after reperfusion, as blood pressure continued to steadily decline over time, even after WB resuscitation (Figure 1).

It is important to acknowledge certain limitations of this study. While heparinization is recognized to influence microcirculation and inflammatory pathways and deviates from clinical practice in shock management, it was necessary to administer heparin to prevent thrombosis within the catheters and pressure transducers for vessel patency and accurate data acquisition. SVR and aortic characteristic impedance were not directly measured (calculated), which minimized invasiveness and perturbations to the model, but would have provided a more comprehensive insight into the hemodynamics following T/HS. Unpublished data suggest marked improvements in preload and cardiac output after enteral protease inhibition, although it is not clear whether this is due to direct cardiac effects or simply a result of more (proteolytically) intact glycocalyx preserving intravascular volume. Despite lack of differences in heart rate between groups, applying mono-exponential fitting to heterogenous waveforms with varying concavity may have led to small inaccuracies in estimating τ, as the method does not account for the effects of wave reflection. Future studies incorporating wave separation analysis (e.g., via direct measurement of reflected wave return time) could offer valuable insights into the distinct contributions of compliance and resistance arteries to waveforms, though these measurements are subject to more variability and methodological complexity. Nonetheless, exponential fitting of the diastolic decay remains a reliable method for the assessment of the Windkessel effect and deriving meaningful data (Vanden et al., 2018), especially when examining relative differences.

Additionally, low-resolution AFM, while fast and convenient, was unable to precisely pinpoint localized regions or vascular components with altered mechanics; therefore, information on the relationship between vascular ultrastructure and biomechanical properties may have been missed. Future studies will investigate the molecular mechanisms affecting altered biomechanics in shock in detail and explore additional humoral or mechanical factors that may contribute alongside serine proteases to the modified AC and the inability to recover DAP after crystalloid-reperfused T/HS.

This study underscores the critical role of arterial compliance in the pathophysiology and management of trauma/hemorrhagic shock. Our hemodynamic and biomechanical analyses revealed that the inability to maintain adequate MAP following T/HS may be caused by disruptions in AC (an often-overlooked component in resuscitation protocols), demonstrating a putative causal link between structural and biomechanical degradation in large artery function and hemodynamic instability following T/HS. The use of enteral serine protease inhibitors demonstrated partial preservation of vascular structure and compliance, effectively stabilizing PWV and improving DAP compared to conventional crystalloid resuscitation alone. However, even with these improvements, whole blood remained superior in achieving optimal hemodynamic stability, likely due to its unique composition and ability to better maintain both vascular and systemic function. Our findings suggest that traditional resuscitation strategies focused solely on volume restoration and vasopressor support may be insufficient, particularly in cases of refractory hypotension, due to the neglect of vascular biomechanics. By elucidating the molecular and structural sources of reduced AC, such as collagen and elastin degradation and increased MMP activity, this study identifies potential therapeutic targets that could mitigate vasoplegia. Targeting the structural and functional modulators of arterial compliance may be an advantageous approach to enhance the effectiveness of T/HS resuscitation, addressing the challenge of persistent hypotension in trauma care and ultimately improving clinical outcomes.

## Data Availability

The original contributions presented in the study are included in the article/Supplementary Material, further inquiries can be directed to the corresponding author.

## Abbreviations

T/HS, Trauma/Hemorrhagic Shock; LR, Lactated Ringer’s; GM, Gabexate Mesilate; WB, Whole Blood; MAP, Mean Arterial Blood Pressure; DAP, Diastolic Arterial Blood Pressure; AC, Arterial Compliance.
