# Supplementary material for: Arterial compliance is improved via enteral serine protease inhibition in experimental trauma/hemorrhagic shock
Source: Front Physiol. 2025 Aug 12;16:1655946. doi: 10.3389/fphys.2025.1655946 (PMC12378548; doi:10.3389/fphys.2025.1655946)
Supplement: Supplementary file 1 [file Supplementaryfile1.docx]

Supplementary Material

# Supplementary Figures and Tables

## Supplementary Tables

**Table S1.** Laboratory parameters of the T/HS groups resuscitated with LR, LR+GM, and WB, including their arterial blood gas profiles at Baseline, at the end of the 90-min hypotension period (Shock), 30 minutes after the start of reperfusion (r30), and 120 minutes after reperfusion (r120).

|  |  | LR | LR + GM | WB | p-value |
| --- | --- | --- | --- | --- | --- |
| Weight (g) |  | 414.3 ± 49.2 | 425.2 ± 28.0 | 425.2 ± 37.7 | 0.6170 |
| Volume Removed (mL) |  | 12.0 ± 1.9 | 12.6 ± 2.6 | 11.5 ± 1.9 | 0.5845 |
| Volume Returned (mL) |  | 19.5 ± 3.9^†,‡^ | 13.8 ± 3.6^*^ | 5.8 ± 1.3 | <0.0001 |
| pH | Baseline | 7.42 ± 0.04 | 7.40 ± 0.06 | 7.41 ± 0.02 | Time = 0.0021  Group = 0.5078  Interaction = 0.1539 |
|  | Shock | 7.46 ± 0.08 | 7.41 ± 0.07 | 7.41 ± 0.07 |  |
|  | r30 | 7.41 ± 0.05 | 7.36 ± 0.06 | 7.42 ± 0.05 |  |
|  | r120 | 7.43 ± 0.09 | 7.49 ± 0.07^§^ | 7.47 ± 0.03 |  |
| pCO_2_ (mmHg) | Baseline | 46.90 ± 5.56 | 50.68 ± 9.12 | 47.63 ± 3.15 | Time < 0.0001  Group = 0.8297  Interaction = 0.6173 |
|  | Shock | 32.89 ± 5.62^§^ | 32.31 ± 8.78^§^ | 34.36 ± 4.45^§^ |  |
|  | r30 | 41.03 ± 3.25 | 43.59 ± 6.79 | 40.91 ± 5.15 |  |
|  | r120 | 38.13 ± 6.71^§^ | 35.53 ± 6.54^§^ | 39.31 ± 3.63^§^ |  |
| pO_2_ (mmHg) | Baseline | 75.91 ± 7.85 | 69.37 ± 12.29 | 73.08 ± 11.15 | Time < 0.0001  Group = 0.0778  Interaction = 0.2669 |
|  | Shock | 107.49 ± 10.56^§^ | 103.34 ± 13.06^§^ | 99.93 ± 11.08^§^ |  |
|  | r30 | 90.57 ± 11.44 | 80.16 ± 11.78 | 84.10 ± 9.73 |  |
|  | r120 | 99.91 ± 16.13^§^ | 105.26 ± 22.82^*, §^ | 88.13 ± 7.51 |  |
| HCO_3_ (mmol/L) | Baseline | 28.24 ± 0.87 | 28.13 ± 1.24 | 28.14 ± 1.08 | Time < 0.0001  Group = 0.0489  Interaction = 0.1489 |
|  | Shock | 23.25 ± 1.40^§^ | 20.89 ± 1.45^§^ | 22.73 ± 3.82^§^ |  |
|  | r30 | 25.20 ± 1.96 | 23.79 ± 2.33^§^ | 26.09 ± 3.29 |  |
|  | r120 | 25.10 ± 3.65^‡^ | 27.07 ± 1.48 | 28.70 ± 1.50 |  |
| BE (mmol/L) | Baseline | 5.49 ± 1.12 | 5.80 ± 1.34 | 5.53 ± 1.1.38 | Time < 0.0001  Group = 0.0862  Interaction = 0.1320 |
|  | Shock | -1.43 ± 1.78^§^ | -4.92 ± 2.08^§^ | -2.55 ± 5.26^§^ |  |
|  | r30 | 1.43 ± 2.43 | -0.64 ± 2.99^§^ | 2.14 ± 4.39 |  |
|  | r120 | 1.14 ± 4.47^‡,§^ | 3.20 ± 1.69 | 5.18 ± 1.86 |  |
| O_2_ sat (%) | Baseline | 87.80 ± 3.25 | 82.75 ± 12.78 | 86.18 ± 6.02 | Time < 0.0001  Group = 0.1104  Interaction = 0.3135 |
|  | Shock | 95.75 ± 2.21^§^ | 95.17 ± 2.25^§^ | 94.64 ± 2.20^§^ |  |
|  | r30 | 94.16 ± 1.92 | 89.67 ± 5.01^§^ | 91.59 ± 2.62 |  |
|  | r120 | 97.50 ± 4.33^§^ | 97.98 ± 3.72^§^ | 93.19 ± 1.75^§^ |  |
| Hemoglobin (g/dL) | Baseline | 14.43 ± 0.64 | 15.17 ± 0.48 | 14.96 ± 0.45 | Time < 0.0001  Group < 0.0001  Interaction < 0.0001 |
|  | Shock | 10.13 ± 1.16^§^ | 9.67 ± 1.68^§^ | 10.04 ± 1.09^§^ |  |
|  | r30 | 8.10 ± 0.54^†,‡,§^ | 9.31 ± 1.44^*,§^ | 11.69 ± 0.57^§^ |  |
|  | r120 | 7.13 ± 0.78^‡,§^ | 8.14 ± 1.13^*,§^ | 11.34 ± 0.72^§^ |  |
| Lactate (mmol/L) | Baseline | 0.86 ± 0.17 | 0.94 ± 0.35 | 0.83 ± 0.29 | Time < 0.0001  Group = 0.0001  Interaction = 0.0411 |
|  | Shock | 5.76 ± 1.46^§^ | 7.60 ± 2.39^§^ | 5.40 ± 3.60^§^ |  |
|  | r30 | 7.46 ± 2.76^‡,§^ | 7.23 ± 3.17^*,§^ | 3.97 ± 2.86^§^ |  |
|  | r120 | 6.00 ± 3.07^‡,§^ | 4.73 ± 1.36^*,§^ | 1.71 ± 0.84 |  |
| Glucose (mg/dL) | Baseline | 254.43 ± 42.40 | 257.33 ± 56.56 | 235.25 ± 27.32 | Time < 0.0001  Group < 0.0001  Interaction < 0.0001 |
|  | Shock | 433.43 ± 56.53^§^ | 492.43 ± 175.84^*,§^ | 362.60 ± 116.01 |  |
|  | r30 | 643.00 ± 75.66^‡,§^ | 582.00 ± 164.80^*,§^ | 289.11 ± 86.34 |  |
|  | r120 | 595.43 ± 155.72^†,‡,§^ | 463.57 ± 111.21^*,§^ | 181.60 ± 33.59 |  |

The p-values by two-way ANOVA are listed for time, group, and interaction factors. * p < 0.05 for LR+GM vs. WB, † p < 0.05 for LR vs. LR+GM, ‡ p < 0.05 LR vs. WB, and § p < 0.05 vs. Baseline for the same group. Abbreviations: pCO_2_ = partial pressure of carbon dioxide, pO_2_ = partial pressure of oxygen, HCO_3_ = bicarbonate, BE = base excess, O_2_ sat = oxygen saturation.

## Supplementary Figures


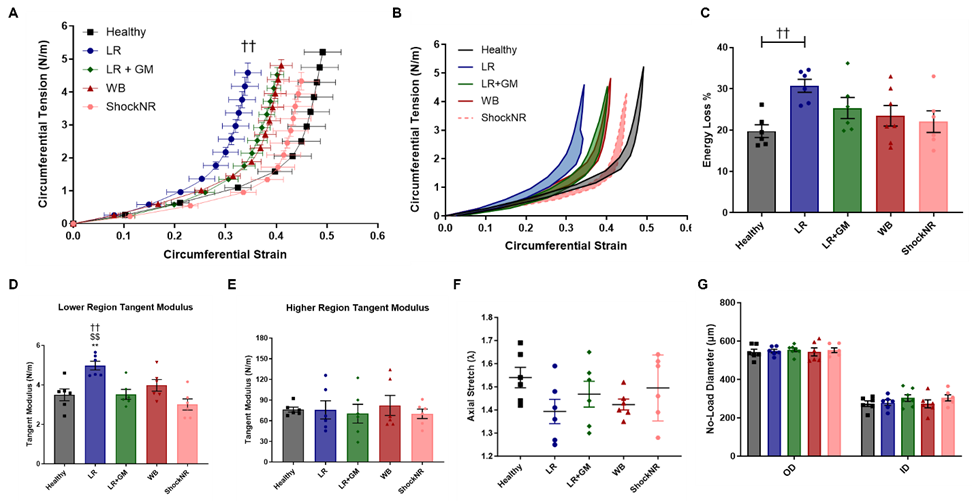


**Figure S1.** Incorporation of a non-resuscitated T/HS group (ShockNR) (n=6) into the ex vivo passive pressure myography data. The biomechanical testing of femoral arteries from an additional ShockNR group (pink) was included for comparison with the original study groups: healthy control (black), untreated LR-resuscitated T/HS (blue), GM-treated LR-resuscitated T/HS (green), and WB-resuscitated T/HS (red). The following data was analyzed: circumferential tension-strain loading curves (A), average hysteresis areas (B), energy loss percentages (C), tangent moduli of the lower linear region (D) and higher linear region (E), axial stretch ratios (F), and zero-load diameters (G). Data are presented as mean ± SEM. By one-way ANOVA, † p < 0.05 and †† p < 0.01 for Healthy vs. LR, ** p < 0.01 for LR vs. ShockNR, and $$ p < 0.01 for LR vs. LR+GM.


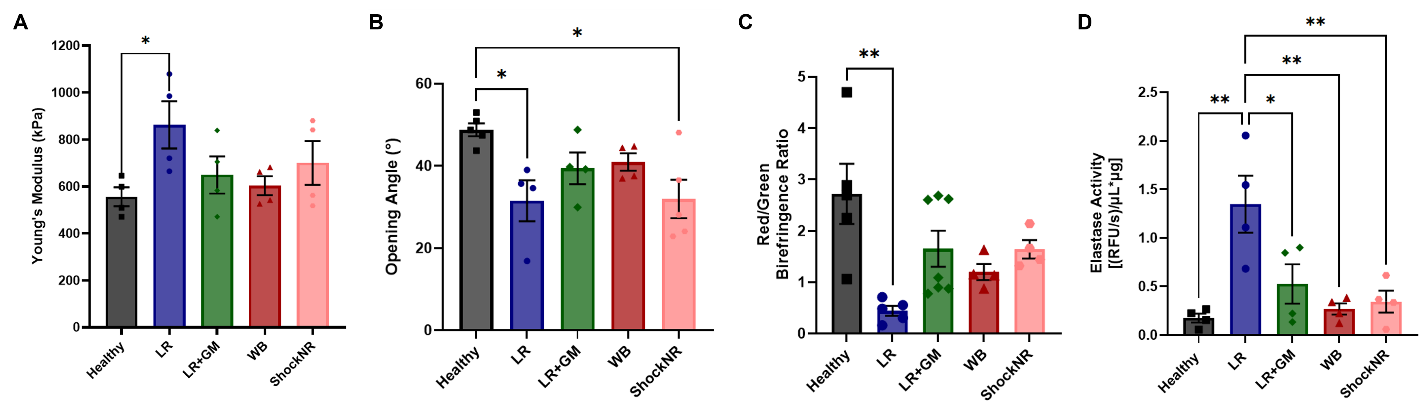


**Figure S2.** Inclusion of a non-resuscitated T/HS group (ShockNR) (n=6) in aortic tissue parameters. ShockNR group (pink) was included for comparison with the original study groups: healthy control (black), untreated LR-resuscitated T/HS (blue), GM-treated LR-resuscitated T/HS (green), and WB-resuscitated T/HS (red). The following data was analyzed: average aortic medial layer Young’s modulus (A), aortic opening angle (B), red/green birefringence ratio via polarized light on picrosirius staining (C), and elastase-like activity in aortic tissue (D). By one-way ANOVA, * p < 0.05 and ** p < 0.01 for marked groups.


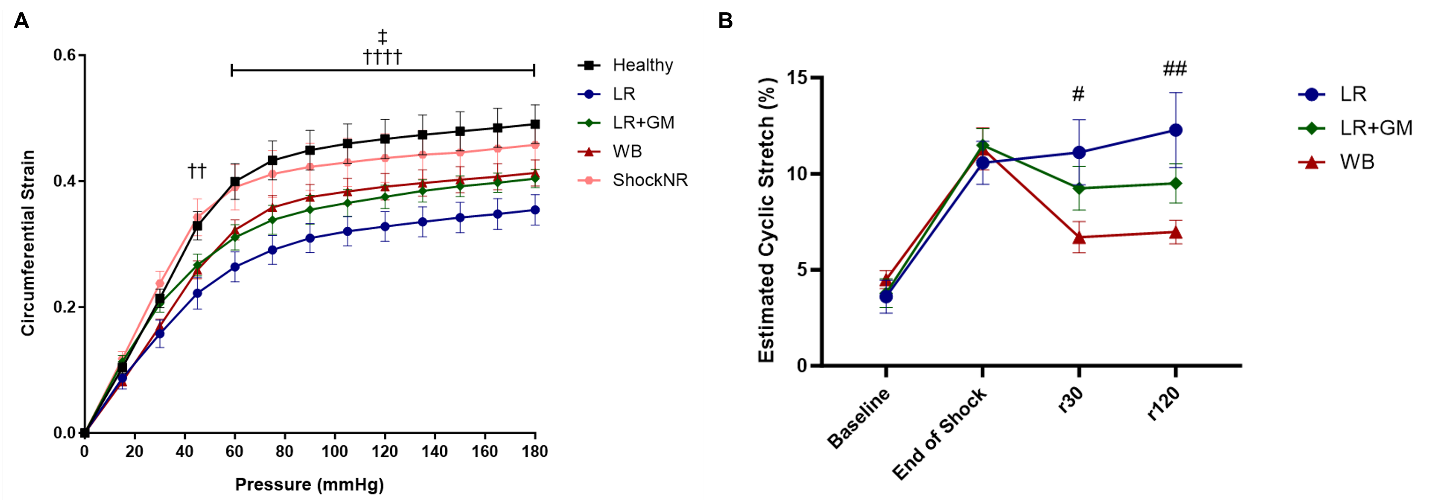


**Figure S3.** Vascular wall deformation at various pressures. Circumferential strains of excised femoral arteries were plotted against its corresponding intraluminal pressure (A) and the non-linear fitting of this data was used to estimate the *in vivo* cyclic stretch experienced at specific timepoints during the T/HS experiment (B). Data are presented as mean ± SEM. By one-way ANOVA, †† p < 0.01 and †††† p < 0.0001 for Healthy vs. LR, ‡ p < 0.05 for Healthy vs. LR+GM, and # p < 0.05 and ## p < 0.01 for LR vs. WB.

**
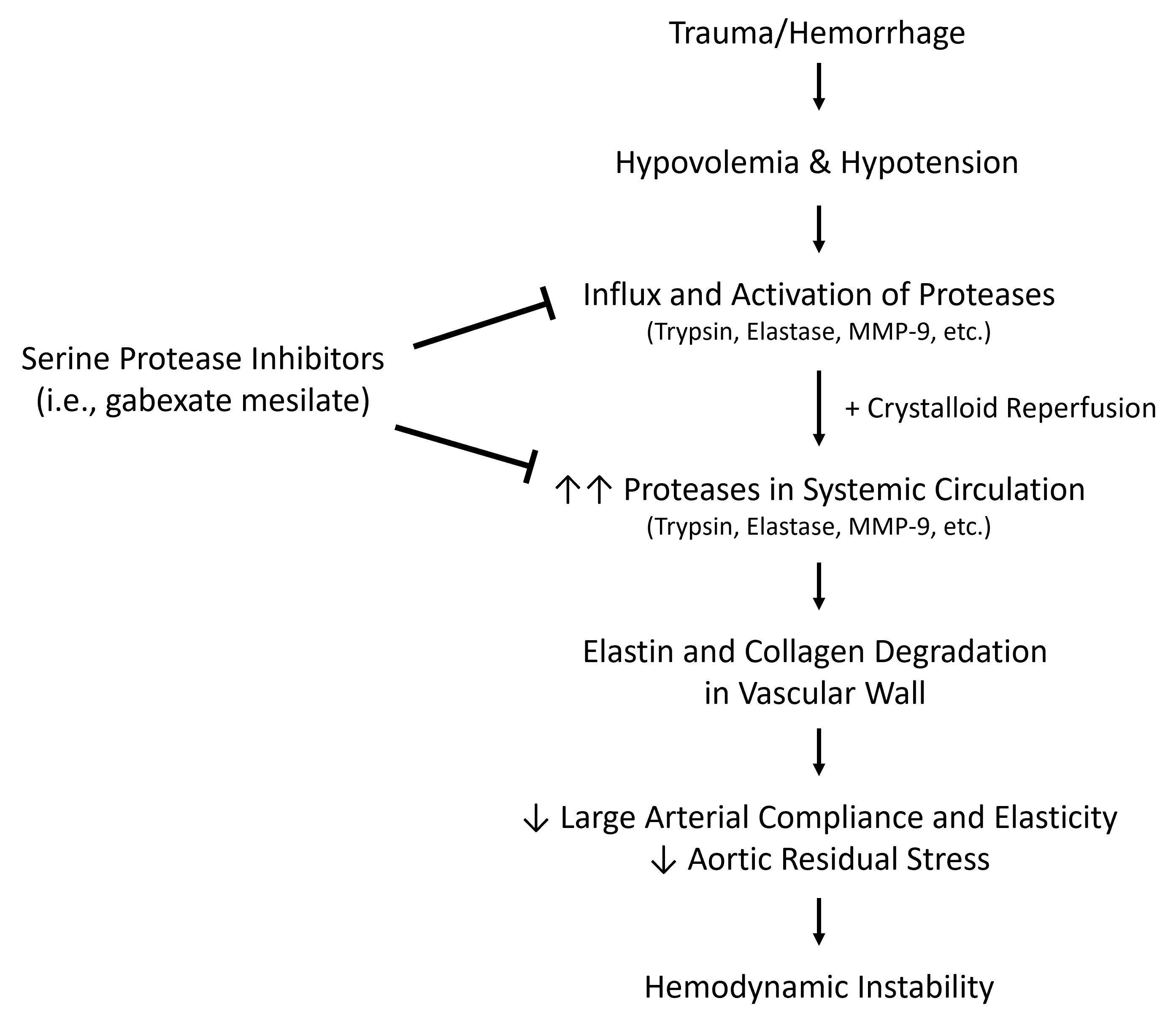
**

**Figure S4.** Schematic diagram of the hypothesized mechanism by which trauma/hemorrhagic shock (T/HS) impairs arterial compliance and hemodynamic stability. T/HS-induced hypovolemia and hypotension leads to the egress of active pancreatic proteases and inflammatory mediators from the ischemic intestine into the circulation. Crystalloid reperfusion may cause additional injury, increasing the concentration of these destructive mediators both systemically and within the tissues. The combined effects of proteolytic enzymes and mechanical stress may lead to elastin and collagen degradation in the vascular wall, comprising the structure and function of large compliance arteries and ultimately contributing to hemodynamic stability.
